# Effect of kynurenic acid on development and aging in wild type and vermilion mutants of *Drosophila melanogaster*

**DOI:** 10.15761/PDDT.1000104

**Published:** 2016-12-08

**Authors:** Valeriya Navrotskaya, Gregory Oxenkrug

**Affiliations:** 1 Department of Genetics and Cytology, V.N. Karazin Kharkiv National University, Ukraine; 2 Psychiatry and Inflammation Program, Department of Psychiatry, Tufts University/Tufts Medical Center, Boston, USA

**Keywords:** kynurenic acid, Drosophila melanogaster, vermilion, aging, development, neurodegenerative disorders

## Abstract

**Background:**

Up-regulation of tryptophan (Trp) conversion into kynurenine (Kyn) and increased formation of down-stream metabolites of Kyn is one of the mechanisms of aging and neurodegenerative disorders. Kyn is an immediate precursor of kynurenic acid (KYNA), an antagonist to NMDA and α7nAChR receptors and activator of aryl hydrocarbon receptor. Increased formation of KYNA ameliorates neurodegeneration and eclosion defect in Drosophila model of Huntington’s Disease.

**Aims:**

Effect of KYNA on pupae viability and life span was evaluated in wild type (Canton-S, CS) and *vermilion* Drosophila mutants with deficient formation of Kyn due to mutation of *vermilion* gene (*v*) that encodes the Trp-2,3-dioxygenase (TDO), enzyme catalyzing Trp conversion into Kyn.

**Methods:**

Vermilion mutants were transferred into the Canton-S genetic background (v-CS). KYNA effect on viability (number of filial generation pupae and %% of their lethality) was assessed in pupae maintained at standard temperature (23°C). KYNA effect on life span was evaluated in adult (imago) flies maintained at 28°C (accelerated aging).

**Results:**

KYNA drastically increased (4 fold from 8.36 to 33.62) %% of dead pupae in Canton-S but not in *v*-CS flies (p=0.0001). KYNA did not affect life span of female Canton-S flies but decreased life span of *v*-CS female flies (from 17.15 to 14.29 days). KYNA increased life span of male Canton-S (from 17.92 to 19.96 days) and v-CS flies (14.52 to 17.75 days).

**Discussion:**

This the first (to the best of our knowledge) observation of the toxic effect of KYNA in Drosophila pupae. KYNA effect on high-temperature induced aging acceleration was gender dependent. Present data support the role of downstream Kyn metabolites in aging mechanisms.

## Introduction

Up-regulation of kynurenine (Kyn) pathway of tryptophan (Trp) metabolism is one of the mechanisms of aging and neurodegenerative disorders, including Huntington’s Disease and schizophrenia [1,2]. Genetic or pharmacologic inhibition of Kyn formation from Trp prolongs life span in *Drosophila* [3–6] and *C. elegans* [3–8]. It was suggested that life-extending effect of inhibition of Trp conversion into Kyn depends on decreased formation of Kyn and its downstream metabolites [2]. Kyn is an immediate precursor of kynurenic acid (KYNA), an antagonist to NMDA and α7nAChR receptors and activator of aryl hydrocarbon receptor [9,10]. Increased formation of KYNA ameliorates neurodegeneration and eclosion defect in Drosophila model of Huntington’s Disease [11,12]. We were interested to evaluate effect of KYNA on life span in Drosophila model. Life cycle of Drosophila consist of pre-imaginal phase (eggs, larvae, pupae) and imago (adult) stage. KYNA effect on life cycle was studied in wild type flies, Canton-S, and in vermilion mutant of Drosophila melanogaster with deficient formation of Kyn from Trp because of mutation of *vermilion* gene (*v*) that encodes the Trp-2,3-dioxygenase (TDO), enzyme catalyzing Trp conversion into Kyn [2]. We assessed KYNA effect on viability of pupae maintained at standard temperature (23°C). Life span of flies is temperature dependent, and flies are living faster at the higher temperature. TDO inhibition attenuates aging accelerated effect of high temperature [13]. So, we evaluated KYNA effect on life span of adult (imago) wild type and vermilion flies maintained at 28°C.

## Methods

Wild-type stock Canton-S of Drosophila melanogaster and mutant stock *vermilion* from the collection of V. N. Karazin Kharkiv National University were used in the experiments. Prior to the experiments vermilion mutants were transferred into the Canton-S genetic background (*v*-CS). The study was carried out between September and December.

Flies were maintained at 23°C in a 12:12 light: dark period on a standard Drosophila medium consisting of sugar, yeast, agar and semolina.

Viability was evaluated by number of filial generation pupae and %% of their lethality as described elsewhere [5,6]. Two pairs of parent individuals (two males and two females) were transferred into a vial containing 4 ml of standard Drosophila medium or medium with addition of KYNA (3 mg/ml) so that so that eggs were deposited directly onto media containing KYNA, and emerging larvae were fed by KYNA throughout development at 23°C.

Nine vials were set up per experimental condition. Number of filial generation pupae and their lethality were calculated per vial.

### Life span evaluation

One day old adult flies were collected and maintained under 28°C to induce accelerated aging [13]. Flies were regularly transferred to fresh medium every 3–4 days. The number of dead flies was recorded at the time of transfer.

### Statistical analysis

Results are presented as mean ± standard error. Statistical significance of differences between experimental groups were assessed by Mann-Whitney test (two tailed).

## Results

### KYNA effect on pre-imaginal development

KYNA did not affect the number of pupae in Canton-S and *v*-CS flies. KYNA increased (4 fold from 8.36 to 33.62) %% of dead pupae in Canton-S flies. KYNA did not affect viability of *v*-CS pupae (Table 1).

### KYNA effect on high-temperature-induced accelerated life span

Life span of flies exposed to high temperature (28°C) was shorter that life span of flies maintained under 23°C in the same laboratory [13,14].

There was no difference in life span between female Canton-S and *v*-CS (16.64 and 17.15 days, resp.) (Table 2).

Life span of male *v*-CS was shorter than of Canton-S male flies (14.52 and 17.92, resp.). KYNA did not affect life span of female Canton-S flies (16.64 and 16.61 days, resp.) but decreased life span of *v*-CS flies (from 17.15 to 14.29 days) (Table 2). KYNA increased life span of both male Canton-S (from 17.92 to 19.96 days) and *v*-CS flies (14.52 to 17.75 days).

## Discussion

There two major findings of the present study. First one is a drastic increase of pupae mortality induced by KYNA administration in wild type (Canton-S) flies. Notably, Kyn pathway of Trp metabolism is initiated during end of larvae phase [15]. Extracts from larvae were reported to have toxic effects on imago, and called paralysins [16]. Some of paralysins were identified as 3-hydroxyKyn and Trp [17]. Present study revealed that larvae products might exert their toxic effects not only at imago but as early as at pupae stage. Although KYNA is considered as protective agent (see Introduction), there are reports of potential toxic effects of KYNA. Thus, prolonged subdural infusion of high doses of KYNA damaged myelin sheaths in the spinal cord of rats and Impaired oligodendrocyte viability [18,19]. These observations are in line with findings of elevated KYNA in brains, spinal fluids and plasma of schizophrenia patients and KYNA-induced impairment of cognition and disruption of pre-pulse inhibition (schizophrenia-like feature) in rats [20–25]. Toxic effect of KYNA in wild type flies might depend on excessive amount of KYNA (endogenously synthesized + added to nutrition medium). Thus, we did not observe negative effect of KYNA on viability of pupae in *v*-CS mutants with deficient KYN formation from Trp (and, consequently, downregulated formation of endogenous KYNA formation from Kyn).

The second finding of the present study is gender-dependent effect of KYNA on high temperature-induced accelerated aging. KYNA extended life span of both wild type and *v*-CS male flies but did not affect life span of wild type and decrease life span of *v*-CS female flies.

It was suggested that that life span extending effect of TDO inhibition depends on suppression of proteotoxicity (due to increased Trp levels) but not on decreased formation of downstream metabolites in the Kyn pathway [8]. Present data suggest contribution of Kyn downstream metabolites to mechanisms of life expending effect of TDO inhibition. Obtained data warrant further studies of gender influences on KYNA effect on life span of flies maintained under normal (23°C) and high (28°C) temperatures.

## Figures and Tables

**Table 1:** Kynurenic acid effect on viability of pupae in wild type and *vermilion* mutants.

Genotype	Control	KYNA

**Canton-S**		
Total number of pupae	58.2 ± 6.34	56.33 ± 5.97
%% of dead pupae (%)	8.36 ± 1.39	33.62 ± 3.69*

***ν*-CS**		
Total number of pupae	48.11 ± 2.3	49.6 ± 4.56
%% of dead pupae	9.84 ± 1.02	11.67 ± 1.7

Data presented as mean ± standard error

* p=0.0001 in comparison with each of experimental group (Mann Whitney two tailed test)

**Table 2:** KYNA and high temperature-induced acceleration of life span of Canton-S and vermilion flies.

Genotype	control	KYNA	P

**Female**			
Canton-S	16.64 ± 0.48 (n=111)	16.61 ± 0.73 (n=71)	Ns
*v*-CS	17.15 ± 0.79 (n=111)	14.29 ± 0.59 (n=116)	0.01

**Male**			
Canton-S	17.92 ± 0.51 (n=108)	19.96 ± 0.73 (n=50)	0.003
*v*-CS	14.52 ± 0.74 (n=100)*	17.75 ± 0.62 (n=76)	0.01

Mean ± standard error days (number of flies)

* p=0.01 in comparison with male Canton-S (Mann Whitney two tailed test)
